# The clinical application of a novel method of internal fixation for femoral neck fractures—dynamic locking compression system

**DOI:** 10.1186/s13018-018-0827-9

**Published:** 2018-05-31

**Authors:** Ya-Ping Xiao, Dong-Ping Shu, Ming-Jian Bei, Tao Ji, Wu-Sheng Kan, Shao-Gang Li

**Affiliations:** 1 Department of Orthopedic Surgery, China Resources & WISCO General Hospital, No. 209 Yejin Road, Wuhan, Hubei Province China; 2 Department of Orthopedic Surgery, Hebei Medical University, Shijiazhuang, China; 3 Department of Orthopedic Surgery, Wuhan Puai Hospital affiliated to Huazhong University of Science and Technology, Wuhan, China

**Keywords:** Femoral neck fractures, Internal fixation, Aged, Surgery, Locking compression plate

## Abstract

**Background:**

Femoral neck fractures are the commonly encountered injury in orthopedic practice and result in significant morbidity and mortality. Currently, how to treat femoral neck fractures safely and effectively is still a challenge. The objective of this study is to evaluate the efficiency of dynamic compression locking system for femoral neck fractures.

**Methods:**

This is a retrospective study conducted from May 2015 to October 2016. The study included 36 patients suffering from femoral neck fractures who underwent closed reduction and were fixed using dynamic compression locking system. All surgeries were performed by the same surgeon. The study was conducted by telephone and on-site follow-up. The Garden classification and anatomical site classification were categorized for all patients. We assessed radiographic outcomes of union, femoral neck shortening, screw back-out, and femoral head avascular necrosis. We also evaluated functional outcome using the Harris hip score. Other outcomes included the length of surgery, duration of hospital stay, injury to surgery time, intraoperative hemorrhage, time to clinical bone union, and other fracture complications.

**Results:**

All patients were followed up 12 to 29 months with an average of 21.58 ± 5.41 months. All cases were caused by falls including 17 males and 19 females with an average age of 65.33 ± 9.30 years old ranging from 53 to 82 years old. Among them, injury to surgery time ranged between 1 and 4 days with an average of 2.58 ± 1.05 days. Duration of hospital stay was 8 to 21 days with an average of 15.33 ± 3.71 days. Intraoperative hemorrhage was 40 to 80 ml with an average of 61.67 ± 12.31 ml. Operation time was from 35 to 80 min with average of 50.25 ± 11.77 min. According to Garden classification, 9 cases (25%) were type II and 27 cases (75%) were type III. According to the anatomical site classification, 8 cases (22.2%) were subcapital femoral neck fractures, 19 cases (52.8%) trans-cervical, and 9 cases (25%) basi-cervical. At present, the follow-up has not found the fracture complications of femoral head avascular necrosis, fracture nonunion, and re-fracture. All patients achieved solid bone union. The mean time of clinical bone union was 3 to 4 months. Among all patients, there were only 3 cases of femoral neck shortening < 5 mm and 1 case of screw back-out = 4 mm. For Harris scoring, average Harris scale at the end of the follow-up was 93.42 ± 3.95 ranging from 85 to 98. There were 32 cases of excellent function scores on the Harris scale and 4 cases of good function scores on the Harris scale. Therefore, the excellent and good rate of Harris hip scores was 100%.

**Conclusions:**

Femoral neck dynamic compression locking system for femoral neck fractures in elderly patients can provide effective stability and reduce complications and revision rates.

## Background

Hip fractures are the most common fragility fractures with the significantly reduced limb function and the markedly decreased quality of life after injuries. The number of hip fractures per annual worldwide is expected to rise to 2.6 million by 2025 and 4.5 million by 2050 [1]. Use of the Garden and anatomical site classification systems has remained the clinical mainstay of femoral neck fracture characterization which help demonstrate appropriate treatment. Operative options include in situ fixation, closed or open reduction with internal fixation, and hip arthroplasty [2].

As a matter of fact, femoral neck fractures are the most common hip fractures, accounting for approximately 57% of all hip fractures [3]. At present, three hollow screws are often used for the internal fixation of femoral neck fractures [4]. It can compress fracture ends, but three hollow screws are not interrelated to form a framework structure. Moreover, the positions of three hollow screws are subjectively influenced by the surgeons. The rotational resistance and vertical shear resistance were poor in some studies [5–7]. Moreover, loosening of internal fixation, displacement of femoral head, and femoral neck nonunion usually happened [8]. During the healing process, lack of effective continuous locking support can lead to the shortening of the femoral neck [5–7]. Femoral neck dynamic compression locking system is a new type of internal fixation for femoral neck fractures and was designed based on the three-dimensional anatomical structure of femoral neck. According to the femoral neck scalene triangle juga structure, the three parallel screws are located on the three jugas, respectively, which are close to the bone cortex [9]. Then, three parallel screws and a lateral pressure locking plate through three locking tail caps integrate into a whole, which forms the stable structure of framework and can precisely and uniformly compress and lock fracture ends with the favorable rotational stability and rigid fixation [10]. This implant was pproved by China medical device regulations (Chinese patent no.: ZL201410458654.9).

Therefore, we retrospectively analyzed patients with femoral neck fractures who were treated with dynamic compression locking system between May 2015 to October 2016 to evaluate the effectiveness, stability, and complications of the dynamic compression locking system.

## Methods

The methodology of our study is a single-center, retrospective study. The institutional review board of China Resources & WISCO General Hospital approved this retrospective study, and informed consents were taken from all the patients. This study assessed patients with femoral neck fractures who underwent fixation with dynamic compression locking system (Suzhou Orthopaedics Instrument CO. Ltd., Suzhou, China) from May 2015 to October 2016. In total, all patients were included and anteroposterior and axial femoral beck radiographs were obtained on first admission following falls. All fracture patterns were recorded according to the Garden and anatomical site classification systems.

All surgeries were performed by the first surgeon. The study was conducted by telephone and on-site follow-up. We assessed radiographic outcomes of union, femoral neck shortening, screw back-out, and femoral head avascular necrosis. Regarding the outcome of femoral neck avascular necrosis, we only assessed the radiographic outcomes of the patients having at least 18 or 24 months postoperatively. We also evaluated functional outcome using the Harris hip scores. Other outcomes included the length of surgery, duration of hospital stay, injury to surgery time, intraoperative hemorrhage, time to clinical bone union, and other fracture complications.

The inclusion criteria were as follows: (1) neck of femur fracture, (2) > 50 years old, (3) closed fractures, (4) the type II and III fractures of the Garden classification, and (5) fractures caused by falling down. Patients who met the above criteria were included in this study. The exclusion criteria were as follows: (1) concomitant ipsilateral femoral shaft fractures, (2) pathological fractures, (3) stress fractures, (4) the type I and IV of the Garden classification, and (5) < 50 years old.

**Fig. 1:**
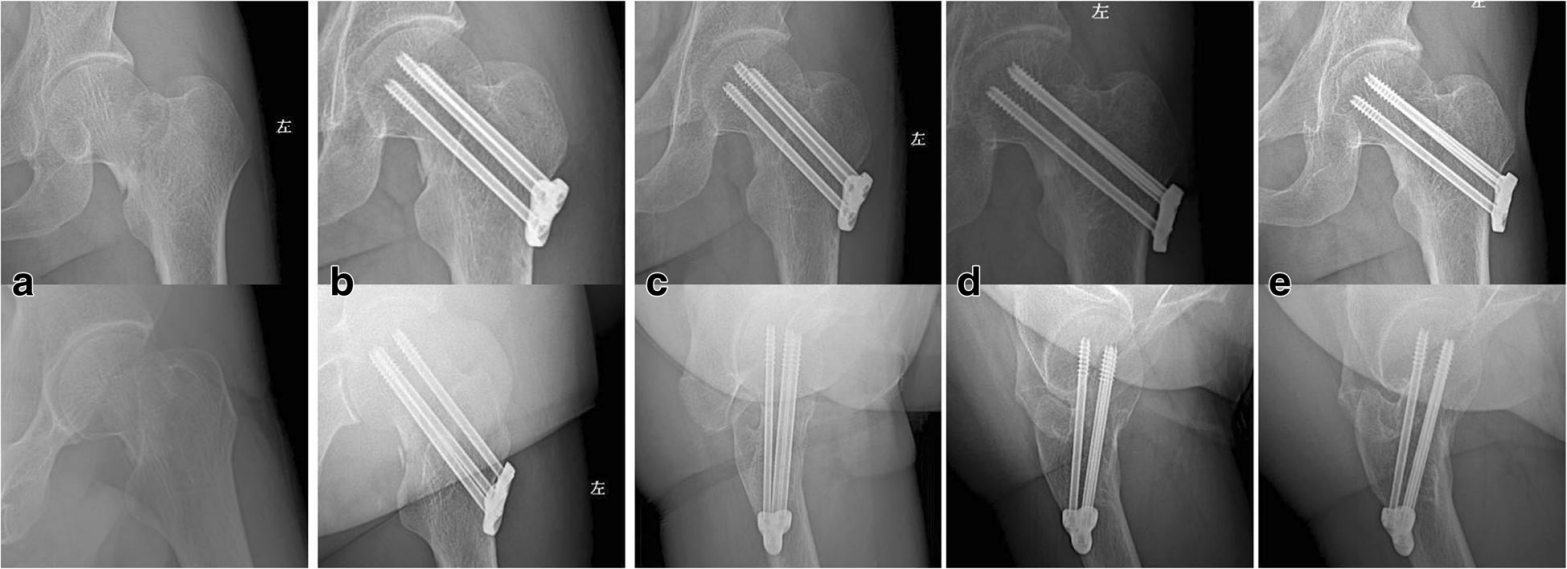
A 54-year-old man with a displaced left femoral neck fracture fixed with dynamic compression locking system. **a** Preoperative X-ray. **b** Postoperative X-ray review at 2 days after operation. **c** Postoperative X-ray review at 1 month after operation. **d** Postoperative X-ray review at 4.5 months after operation. **e** Postoperative X-ray review at 18.5 months after operation

In this study, 36 cases with femoral neck fractures were caused by falls including 17 males and 19 females, with an average age of 65.33 ± 9.30 years old ranging between 53 and 82 years old (Fig. 1). Among them, injury to surgery time was 1 to 4 days with an average of 2.58 ± 1.05 days. Duration of hospital stay was 8 to 21 days with an average of 15.33 ± 3.71 days. Intraoperative hemorrhage was 40 to 80 ml with an average of 61.67 ± 12.31 ml. Operation time was 35 to 80 min with an average of 50.25 ± 11.77 min. According to the Garden classification standards, type II fractures were seen in 9 cases and type III 27 cases. According to the anatomical site classification, subcapital femoral neck fractures were found in 8 cases, trans-cervical fractures of femur 19 cases, and femoral neck basal fractures 9 cases.

Of the patients, 16.7% had diabetes mellitus, 44.4% had hypertension, 33.3% had ischemic heart disease or a previous cerebrovascular event, 22.2% had pulmonary diseases, and 27.8% had hyperlipidemia.

### Surgical procedure

**Fig. 2:**
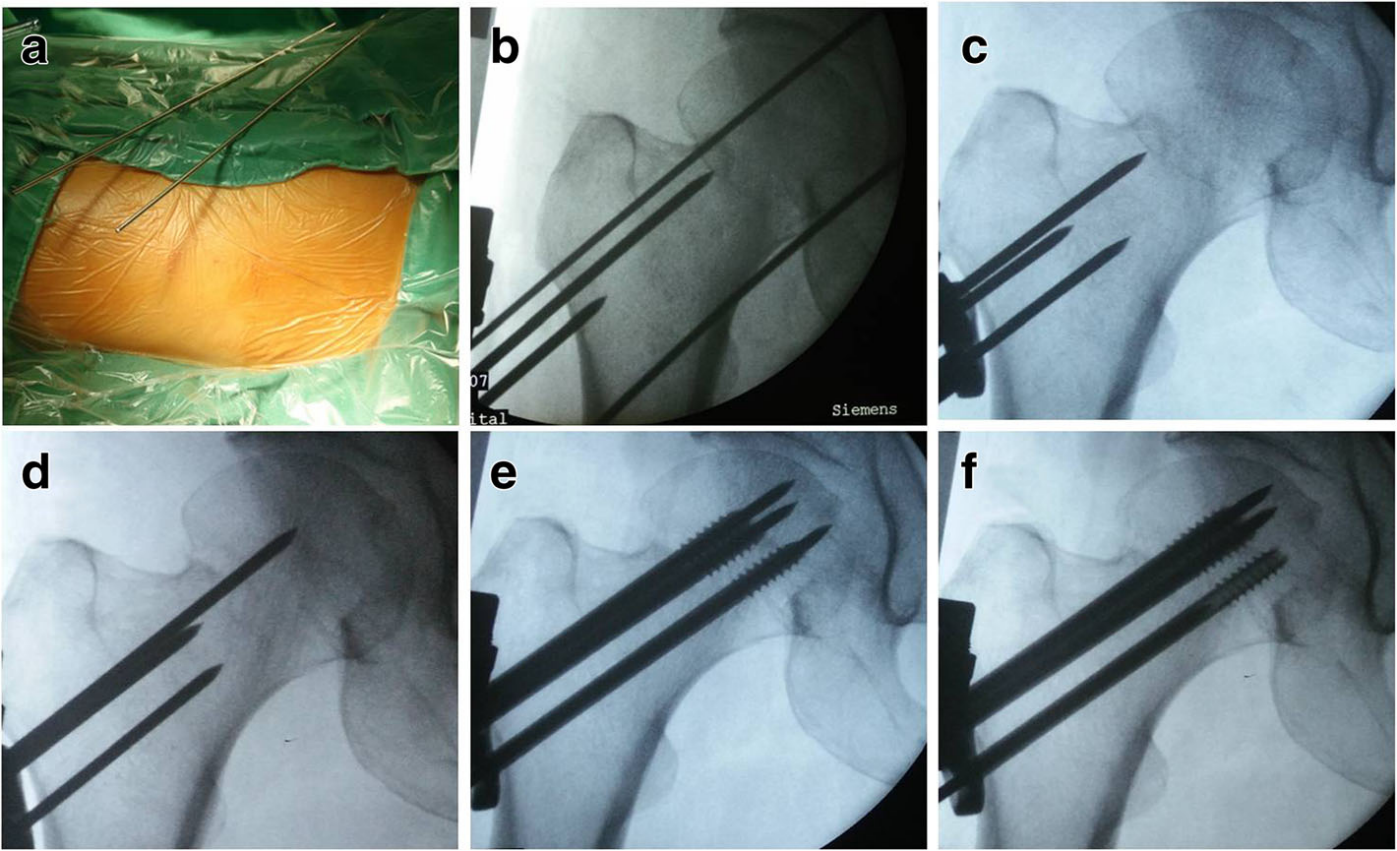
Surgical procedure of the dynamic compression locking system for femoral neck fracture was conducted by the same surgeon. **a** Placement of extracorporal guide needles. **b** Insert the top guide needle into the femoral neck along the direction of the extracorporal guide needles. **c** Install the guide sleeve and insert two other guide needles along the direction of the top guide needle. **d** After the fracture reduction by closed reduction, the top guide needle was transferred to the femoral head to maintain the repositioned position. **e** Insert three hollow parallel compression screws along the direction of the guide needles. **f** Pressure screws evenly and accurately

After anesthesia involving a continuous epidural or lumbar anesthesia, the patients were supine with the contralateral leg flexed at 90° to enable visualization of the fracture. Then, routine disinfection and sterile draping of the hip and lower extremities was carried out. The lateral minimal incision over the greater trochanter of the femur was performed with a 4–5-cm incision length, then the superficial and deep fascial layers and muscles were isolated sequentially, and the lateral cortex of the large tuberosity of the femur was exposed to 6–7 cm length. One or two extracorporeal guide needles were placed on the hip. The position of the extracorporeal guide needle was adjusted until the needle was parallel to the longitudinal axis of the femoral neck. According to the direction of the extracorporeal guide needle, surgeon can insert the top guide needle into the anterior and superior position of the greater trochanter of the femur with the continuing monitor of C-arm radiographs. And then along the top guide needle, surgeon can install locking plate that had installed guide needle sleeve in the greater trochanter of the femur. In the direction of the guide sleeve, surgeon can place the other two parallel guide needles along the direction of the top guide needle into distal fracture end, and finally the three parallel guide needles construct an scalene triangle. A closed reduction of femoral neck fracture was observed with the monitor of C-arm fluoroscopy. To the Garden III fractures [11], ahead of adduction and internal rotation, outreach and extorsion of axial traction were conducted and until closed reduction of the fracture ends was satisfied with the dynamic monitoring of anteroposterior and axial radiographs. Then, surgeon can roll the three parallel guide needles into femoral head within 5 mm of the subchondral bone of the femoral head. The required lengths of the needles were measured to guarantee the satisfying location of the screw placement. All three screws were positioned within 5 mm of the subchondral bone of the femoral head. Finally, surgeon rolled three parallel hollow locking compression screws through the direction of the three guide needles respectively into the neck of femur. At the end of tightening screws, three parallel screws(7 mm) should be uniformly and accurately compressed. When the intraoperative radiographs suggested that compression fixation of the fracture ends was satisfying, the tail caps were tightened into the end of the three screws. In the end, sealing the incision layer-by-layer should be prudentially conducted (Fig. 2).

### Postoperative management

Postoperatively, patients were discharged 1–3 week after surgery. Patients with ischemic heart disease or a previous cerebrovascular event were treated with anticoagulants for 14 days. A wound check was observed by 2- to 3-day intervals till the removing of the suture 2 weeks after surgery. Follow-up by X-ray is done at 1 week after surgery and monthly intervals till complete union was achieved, then at 6 months’ intervals till final follow-up. Hip range was encouraged as soon as tolerated by the patient (usually few days after surgery); partial weight bearing was started when tolerated by patients. Full weight bearing was allowed only when full union was achieved.

All patients were followed up for at least 1 years postoperatively. Close follow-up at our outpatient department included radiographs of the femoral neck to assess fracture healing and the recording of functional outcomes using the Harris hip scores. All outcomes of patients were assessed by two blinded observers.

## Results

No patients had died within the last follow-up. All patients were followed up about 12 to 29 months with an average of 21.58 ± 5.41 months. At present, the follow-up has not found the fracture complications of femoral head avascular necrosis, fracture nonunion, and re-fracture. There were no reoperation and hip joint contracture and difficulty within the final follow-up. All patients achieved solid bone union. The time to bone union was 3 to 4 months. Among all patients, we used the method adopted by Zlowodzki [12] to evaluate femoral neck shortening and we found that only 3 cases (8.3%) had shortening < 5 mm and were considered as non/mild. Only 1 case screw back-out distance was 4 mm. For Harris scoring [13], average Harris scale at the final follow-up was 93.42 ± 3.95 ranging from 85 to 98.According to the function score of Harris hip, the score of 90–100 is divided into excellent, 80–89 is divided into good, 70–79 is fair, and 70 points below is poor. There were 32 cases of excellent function score of Harris hip and 4 cases of good function score. Of the latter, 3 cases had femoral neck shortening < 5 mm. Therefore, the excellent and good rate of the Harris hip scores was 100%. There were no perioperative complications including deep vein thrombosis, pulmonary embolism, urinary tract infection, wound hematoma, and bleeding gastrointestinal tract probably due to the effect of early straight leg raising training on postoperative rehabilitation of patients (usually 2 to 3 days after surgery).

## Discussion

After femoral neck fractures, no matter what type of treatment is chosen, it will have a significant impact on the quality of patients’ life and bring a great economic burden to the society [4]. Compared with hip joint replacement, internal fixation surgery, because of its smaller trauma, shorter operation time, less bleeding, lower incidence of postoperative complications and early mortality, and lower cost, has become the main treatment of femoral neck fractures clinically [4]. However, there is still lack of a consistent view of what methods of internal fixation can better maintain the stability of fracture ends, promote fracture healing, and avoid or reduce the complications such as postoperative femoral head avascular necrosis and internal fixation failures [14].

In our study, longer duration of hospital stay was associated with the presence of ischemic heart disease, cerebrovascular accident, and pulmonary disease, as these patients needed to be optimized before discharge. Nonetheless, advanced age did not significantly affect the duration of hospital stay. In other studies, duration of hospital stay was affected by the number of comorbidities but not advanced age [15]. The lateral minimal incision over the greater trochanter of the femur was performed with a 4–5-cm incision length. Therefore, smaller trauma with a smaller amount of blood loss can effectively reduce soft tissue exposure and ultimately benefits fracture healing. Surgery was performed early (an average of 2.58 ± 1.05 days after injury) in our hospital to reduce the complications associated with post-fracture bedridden, thereby increasing the surgical tolerability. Moreover, early hip joint function exercise after operation effectively reduced postoperative complications. As a result, perioperative complications did not occur in this study. Our study shows lesser incidence of perioperative complications compared with other studies [16].

**Fig. 3:**
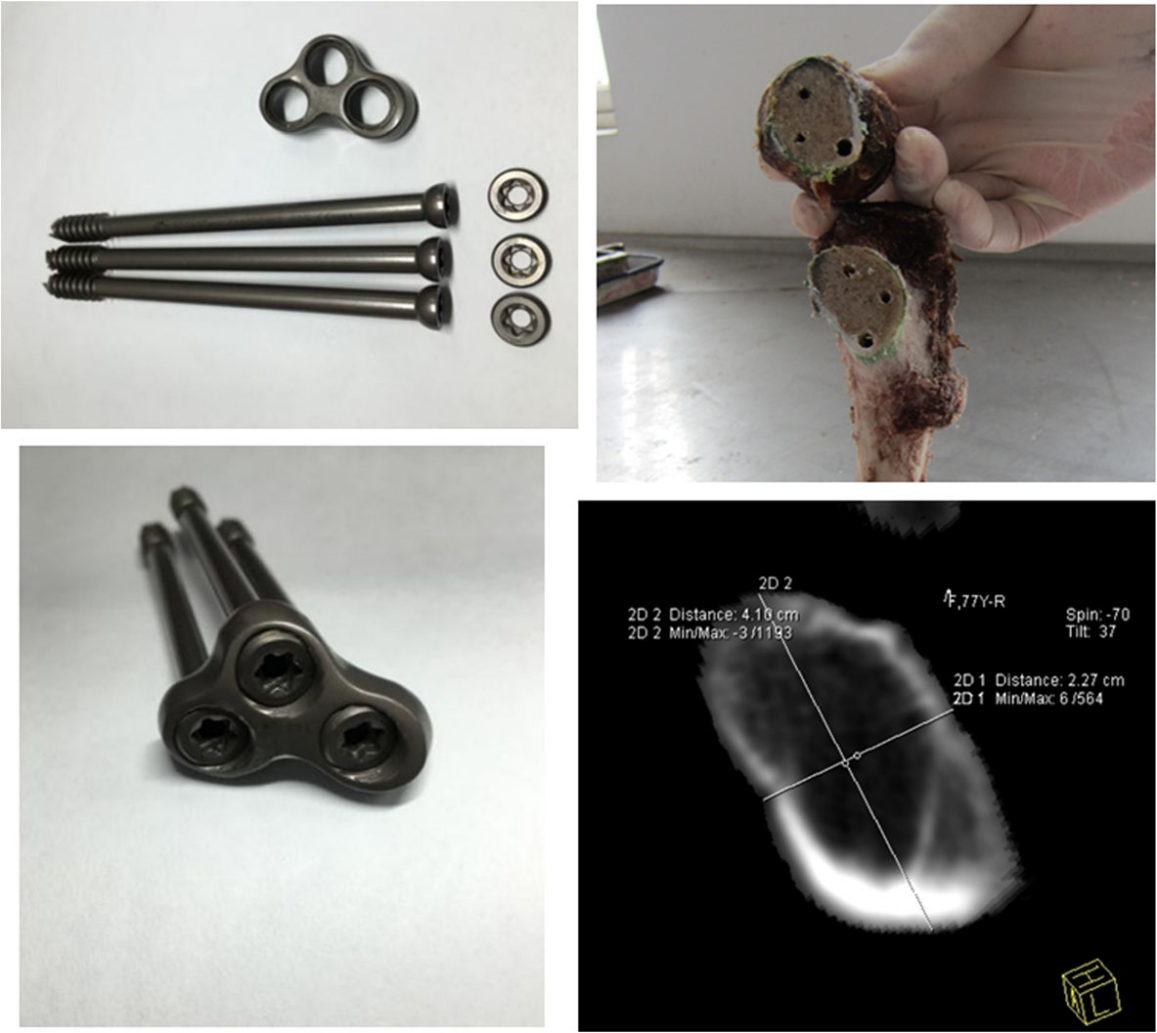
The composition of the dynamic compression locking system and the inverted triangular structure or triangle juga structure of femoral neck obtained by the cadaver model or the cross-section computer software

Our researchers used computed tomography technique to study the anatomical features of femoral neck. The analysis of femoral neck structure using 3D imaging and the cross-section computer software revealed that the cross-section of femur neck was an scalene triangle configuration [9]. “Femoral neck safety cross section” was obtained by the cross-section computer software, so the scalene triangular distribution of three screws in femur neck was developed. The three parallel screws are located in the three jugas of the scalene triangle configuration and are close to the bony cortex, which are excellent in biological stability [10]. A lateral locking plate and three interlocking tail caps are used to interlink three hollow compression screws into a whole to provide rigid fixation, which constitutes a dynamic compression locking system (Fig. 3). This system can not only improve the precision and uniform pressure of fracture ends, but also have a stable framework structure to lock fracture ends. Moreover, the system is capable of compressing fracture ends dynamically [10]. And ultimately, the ends of the fracture can obtain good short- and long-term fixation, and the rigid fixation ultimately is conducive to fracture healing. Early biomechanical studies have found that the finite element analysis of this system in the cadaver model was close to the biomechanical conduction of the normal femoral neck with no stress occlusion [10], so this system is conducive to the growth and healing of bone scabs in the fracture area. Currently, the follow-up has not found the fracture complications of femoral head avascular necrosis, fracture nonunion, and re-fracture. There were no reoperation and hip joint contracture and difficulty at the final follow-up. All patients achieved solid bone union. But, 3 cases (8.3%) had femoral neck shortening < 5 mm and 1 case had screw back-out distance = 4 mm, which was associated with severe osteoporosis or sequela of cerebral infarction. In our study, all patients with femoral neck fractures were caused by falls with good preoperative mobility. So no patients had died within the final follow-up. Other studies reported that the mortality rate was associated with preoperative mobility, and preoperative mobility was considered the most significant determinant for postoperative survival [16].

The most commonly internal fixation for fixing femoral neck fractures is three cancellous screws [4, 7, 8, 16]. Recent studies described different techniques for divergent screw fixation of neck of femur fractures with the different distribution and direction of the screws in femoral neck [4, 7, 8, 16]. The rotation stability of the three cancellous screws was relatively variable, and ultimately, the instability of fracture ends in some studies often adversely influenced the fracture healing [17–19]. However, the distribution of the three interlocking compression screws of this system in femoral head position was according to the principle of anatomy and biomechanics of the femoral neck. One is located in the anterior and inferior part of the femoral neck with longitudinal pressure, another one sits in the anterior superior tension side of the femoral neck, and the last one is in the rear and slightly inferior position of the femoral neck. The fixed positions of the screws are the position in femoral neck with the highest bone density [9]. This system provides rigid fixation so that the anti-rotation resistance is significantly enhanced. The finite element mechanics analysis of this system and the three cannulated screws for femoral neck fractures in the cadaver model suggested that the maximum stress of the three screws was greater in the three cannulated screws than in this system (138.9 vs. 57.49 MPa) [9, 10]. The contact pressure of fracture surface was higher in the three cannulated screw model than in this system model (46.9 vs. 22.58 MPa) [9, 10]. Moreover, the three screws in the femur were more stressed in the three cannulated screw model than in this system model. The greater the stress value, the greater the damage to the screws, which can cause the screw to break. Therefore, the impact on the normal femoral stress conduction and stress shelter are smaller in this system compared with the three cannulated screws. In the case of a tenfold amplification of the fissure of contact surface, the three cannulated screws had the larger fissure than this system had [9, 10]. The wider fissure with the relatively terrible contact surface of fracture causes serious damage to the healing of fracture. Consequently, the more stable fracture ends in this system compared with the three cannulated screws is conducive to fracture healing. Weil et al. [20] assessed femoral neck shortening after internal fixation of femoral neck fractures in 41 patients fixed with three cannulated screws in inverted triangle configuration. He found that femoral neck shortening (> 5 mm) occurred in 56% of the patients and severe shortening (> 10 mm) in 22% of patients. Screw backing out (> 5 mm) occurred in 17 (41%) patients. Seven patients required late (> 6 months after primary internal fixation) arthroplasty. In our study, only 3 cases (8.3%) had shortening < 5 mm and 1 case had screw back-out distance = 4 mm.

**Fig. 4:**
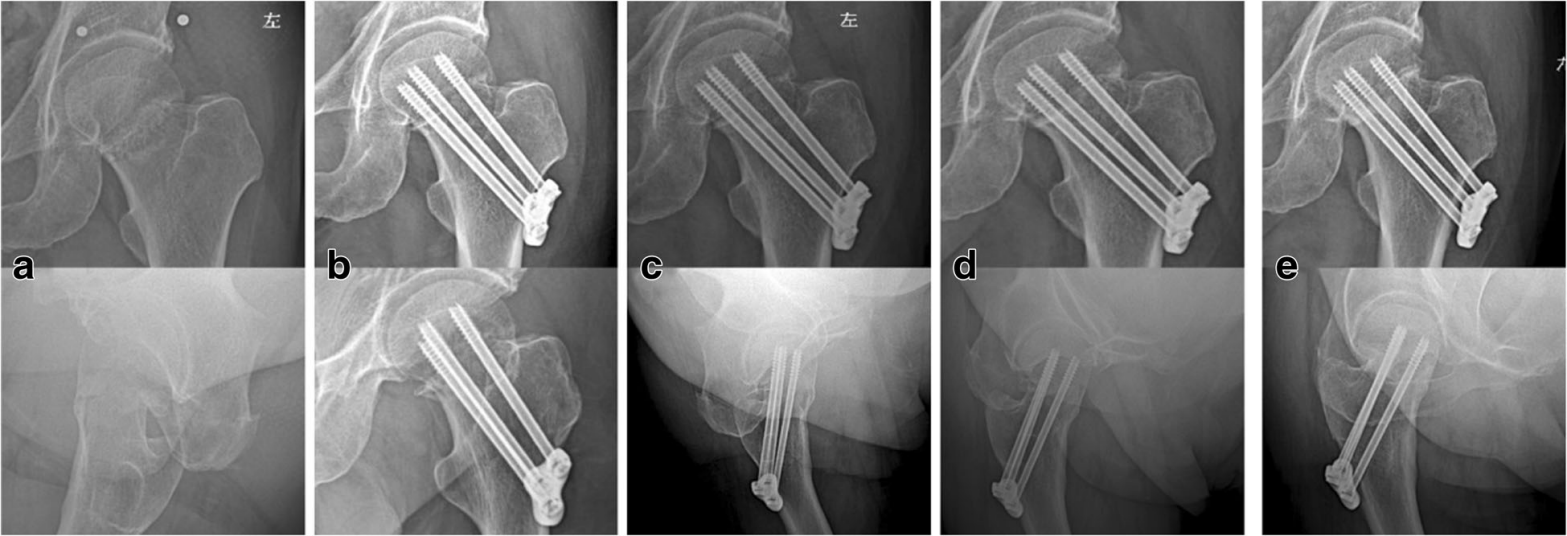
Radiograph showing slight screw back-out. A 67-year-old man fixed with dynamic compression locking system without the irritation of the soft tissue around the plate. **a** Preoperative X-ray. **b** Postoperative X-ray review at 1 day after operation. **c** Postoperative X-ray review at 2 months after operation. **d** Postoperative X-ray review at 8 months after operation. **e** Postoperative X-ray review at 20 months after operation

The design and mechanism of dynamic compression locking system for fixing femoral neck fractures are very similar to these of the Targon FN implant, both of which provide an angular and rotator stable construct and offer a unique sliding mechanism, allowing for controlled fracture impaction. However, the Targon FN implant is combined with two distal locking screws, which can improve rotational and angular stability, but weaken the ability of sliding compressive mechanism [21]. Nishiyama et al. [22] indicated that a sliding mechanism allowing linear intraoperative and postoperative compression on the treatment of femoral neck fractures facilitated fracture healing. The Targon FN is designed to give a better rotational and angular stability than other contemporary internal fixation devices used for femoral neck fractures [21]. Osarumwense et al. [23] showed that the results of the Targon FN system for the management of intracapsular neck of femur fractures in a study with minimum 2-year experience and outcome were superior to those then found in the literature for the more traditional fixation methods. Nonunion rates are reported to range between 2.2 and 2.7% after undisplaced fractures and even 2.2 and 15.4% after displaced fractures [24–26]. Several studies reported a rate of 4–5.5% of avascular necrosis after Targon FN fixation of undisplaced fractures and 4–13.8% after displaced fractures [21, 27]. Parker and Takigawa et al. [21, 27] reported elective implant removal in 10.9% after undisplaced fractures and in 6.4–48.3% after displaced fractures, also mainly due to discomfort. Besides, Biber et al. [24] reported that hematomarate of the internal fixation for femoral neck fracture fixed by Targon FN was 4.4% (95% CI, 0.9–8.0) and cutout rate was 9.6% (95% CI, 4.6–14.7). Peri-implant fracture was 1.2% [21]. In our study, the follow-up currently has not found the fracture complications of femoral head avascular necrosis, fracture nonunion, hematomarate, and re-fracture. Among all patients, there was one case of screw back-out = 4 mm without the irritation of the soft tissue around the plate (Fig. 4). Our results compare favorably with the results of the Targon FN, which warrants further prospective evaluation of multicenter, large sample randomized controlled clinical study.

## Limitations

This study had several limitations involving its retrospective nature and small sample size. Postoperative radiographs and Harris hip scores of patients were evaluated at different timelines. In addition, there was a lack of a control group of patients treated with three cannulated screws.

## Conclusion

Dynamic compression locking system represents a reliable method of fixation for femoral neck fractures. The main prerequisite for the proper healing of femoral neck fractures with this method is stable and rigid fixation. Besides, this method offers a unique sliding mechanism, allowing for controlled fracture impaction. So this way of fixation allows operated limb to have the early hip functional training in optimal time. At present, dynamic compression locking system is in the preliminary stage of clinical application, and the follow-up effect is satisfactory. It is worth promoting its use in clinical practice and continuing to study its clinical effect in the second stage.
